# Case report: Tirzepatide-associated morbilliform drug eruption and implications for rising glucagon-like peptide-1 agonist use

**DOI:** 10.1016/j.jdcr.2026.06.006

**Published:** 2026-06-12

**Authors:** Dorothy S. Peng, Amy Shen, Chandra Smart, Jayden Galamgam

**Affiliations:** a David Geffen School of Medicine at University of California Los Angeles, Los Angeles, California; b Division of Dermatology, Department of Medicine, David Geffen School of Medicine at University of California Los Angeles, Los Angeles, California; c Division of Dermatopathology, Department of Pathology and Laboratory Medicine, David Geffen School of Medicine at University of California Los Angeles, Los Angeles, California

**Keywords:** cutaneous adverse drug reaction, drug hypersensitivity, dual GLP-1/GIP agonists, eosinophilic spongiotic dermatitis, exanthematous drug eruption, GLP-1 receptor agonists, morbilliform drug eruption, pharmacovigilance, systemic therapy, tirzepatide, type IV hypersensitivity

## Introduction

Glucagon-like peptide-1 (GLP-1) receptor agonists and dual GLP-1/glucose-dependent insulinotropic polypeptide (GIP) receptor agonists have become major therapeutic options for type 2 diabetes and obesity management. Tirzepatide, the first dual GLP-1/GIP receptor agonist, received FDA approval for type 2 diabetes in 2022 and for obesity in 2023.^1^ Use of this drug class has expanded rapidly: an analysis of the TriNetX database showed that among a cohort of approximately 45 million U.S. patients, those initiating GLP-1 agonist therapy rose from 27,367 in 2011-2014 to 679,265 in 2019-2023,^2^ representing nearly a 25-fold rise in under a decade.

Despite their growing use, serious cutaneous adverse effects remain rare. A 2025 systematic review identified only 33 patients across 31 studies with GLP-1 agonist-associated cutaneous reactions, most commonly dermal hypersensitivity (33.3%) and eosinophilic panniculitis (30.3%), followed by bullous pemphigoid, angioedema, and morbilliform drug eruptions.^3^ In the SURPASS trials, hypersensitivity reactions were reported in approximately 1% to 2% of tirzepatide-treated patients, all mild to moderate and none resulting in drug discontinuation.^4^ Only 2 cases of morbilliform drug eruption linked to dulaglutide have been reported.^5^^,^^6^

Morbilliform eruptions, also known as exanthematous drug eruptions, represent the most frequent form of cutaneous adverse drug reaction.^7^ Clinically, they present as erythematous macules and papules that may coalesce into patches, typically arising 5-14 days after drug initiation.^7^ These reactions are mediated by type IV hypersensitivity, specifically type IVa and IVb subtypes, in which T cells recognize drug antigens and release cytokines that recruit inflammatory cells, including eosinophils.^8^ Here, we report a case of tirzepatide-associated morbilliform drug eruption.

## Case

**Fig 1:**
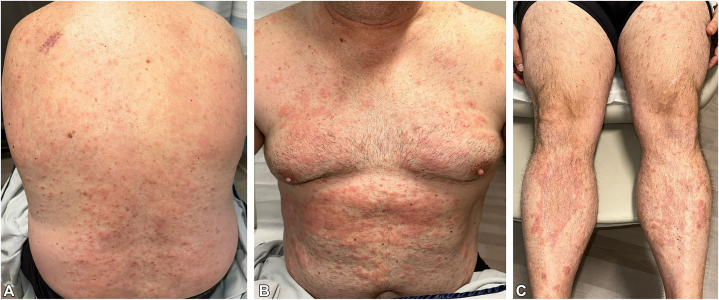
Tirzepatide-associated drug eruption presenting as a diffuse pruritic eruption with erythematous, scaly papules on the **(A)** back, **(B)** chest, and **(C)** legs.

A 55-year-old man with type 2 diabetes mellitus, whose only medications were lisinopril for many years and tirzepatide for 1 year, developed a diffuse pruritic eruption with erythematous, scaly papules on the trunk and extremities (Fig 1). The first biopsy favored an eczematous process with follicular involvement, and topical triamcinolone was started. One month later, the eruption worsened; a second biopsy demonstrated superficial and deep perivascular lymphocytic infiltrate with scattered dermal eosinophils. The patient continued topical triamcinolone for 6 weeks without improvement and was referred to a tertiary dermatology center for further evaluation.

A third biopsy demonstrated similar findings, prompting initiation of oral doxycycline for 1 month to target a possible follicular component, along with alternating topical triamcinolone and tacrolimus. When no improvement was observed, a 4-week prednisone taper was added; however, the eruption persisted. Given that lymphomatoid papulosis was also considered based on the clinical morphology (despite the absence of epidermotropism on histopathology), methotrexate was trialed for 6 weeks without benefit. A fourth and final biopsy revealed eosinophil-rich spongiotic dermatitis. At that time, direct immunofluorescence was performed as the differential diagnosis had broadened to include urticarial-phase bullous pemphigoid. The direct immunofluorescence study was negative, making bullous pemphigoid less likely. No vesicles or bullae developed during the clinical course.

Laboratory studies, including complete blood count, metabolic panel, and liver function tests, remained within normal limits throughout the course, and no peripheral eosinophilia was observed. Given the severity and persistence of disease, as well as the eosinophil-rich spongiotic dermatitis seen on histopathology and the eczematous clinical morphology, dupilumab was initiated. However, 3 weeks after starting dupilumab, the patient noticed a flare of the eruption following his routine tirzepatide injection, and based on the temporal correlation between drug administration and symptom exacerbation, tirzepatide was discontinued.

**Fig 2:**
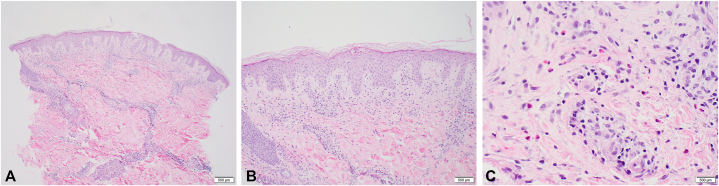
Biopsy of tirzepatide-associated drug eruption. Hematoxylin and eosin-stained sections show **(A)** Low power view demonstrating spongiotic dermatitis with underlying, superficial/deep, perivascular/adnexal inflammation (original magnification ×40). **B,** High power view demonstrating epidermal spongiosis and intraepidermal collections of Langerhans cells (original magnification ×100). **C,** High power view demonstrating a dermal inflammatory infiltrate composed of lymphocytes, histiocytes, and many eosinophils (original magnification ×400).

Following cessation of tirzepatide, the patient experienced complete resolution of the morbilliform eruption with a brief 1-week prednisone taper and continuation of topical triamcinolone. The dramatic improvement upon drug withdrawal, combined with the temporal relationship and characteristic histopathological findings, suggested a tirzepatide-induced morbilliform drug eruption. The eosinophil-rich infiltrate observed in our patient's final biopsy (Fig 2) is consistent with a type IVb hypersensitivity mechanism, supporting the diagnosis of a drug-induced morbilliform eruption.

## Discussion

A distinctive feature of this case is the delayed onset of eruption after more than a year of continuous tirzepatide use. By contrast, the 2 previously published dulaglutide-associated cases occurred within 5 weeks of initiation. One describes an 84-year-old man who developed a widespread morbilliform eruption with purpuric discoloration and petechiae,^5^ and the other a 75-year-old man with a similar presentation.^6^ Both resolved following drug discontinuation and systemic corticosteroid therapy. This extended latency is unusual, as most drug eruptions arise within 5-14 days of exposure.^7^ However, it is biologically plausible given the variability of type IV hypersensitivity reactions, which may manifest after prolonged antigenic stimulation.^8^

This case highlights several clinical management principles. First, the diagnosis of drug-induced morbilliform eruption relies heavily on temporal correlation between drug administration and symptom onset or exacerbation. Second, histopathologic examination is supportive but not definitive, as features may evolve from nonspecific interface or eczematous patterns to an eosinophil-rich spongiotic dermatitis more typical of hypersensitivity. Third, drug cessation remains the cornerstone of treatment, with corticosteroids, antihistamines, or biologics as adjuncts.

The clinical course demonstrated refractoriness to multiple therapeutic interventions, including topical corticosteroids, systemic corticosteroids, doxycycline, methotrexate, and biologics, during continuous tirzepatide exposure. This treatment resistance is consistent with ongoing antigen exposure maintaining the hypersensitivity reaction.^9^ The dramatic improvement following drug cessation provides compelling evidence for the causative role of tirzepatide and emphasizes the critical importance of drug withdrawal in managing suspected cutaneous adverse drug reactions.

Tirzepatide utilization has grown substantially,^1^ amplifying the clinical significance of recognizing dermatological adverse effects of GLP-1 and GLP-1/GIP receptor agonists. While SURPASS trials documented hypersensitivity reactions in approximately 1% to 2% of tirzepatide recipients, these were limited to mild to moderate injection-site reactions with no progression to morbilliform eruption.^4^ The delayed onset observed in this case highlights that clinicians should maintain vigilance for cutaneous adverse reactions throughout the duration of therapy, not only during initiation.

## Conclusion

This case adds to the literature on GLP-1/GIP receptor agonist-associated cutaneous adverse reactions. Clinicians should consider the possibility of a drug-induced morbilliform eruption in patients presenting with generalized rashes while receiving GLP-1 pathway therapies, even after long-term exposure, as hypersensitivity reactions may arise with variable latency.

## Conflicts of interest

None disclosed.

## References

[1] Hankosky E.R., Chinthammit C., Meeks A. (2025). Real-world use and effectiveness of tirzepatide among individuals without type 2 diabetes: results from the Optum Market Clarity database. Diabetes Obes Metab.

[2] Yeo Y.H., Rezaie A., Hsieh T.Y.-J., Hu X., Gaddam S., Ma K.S.-K., Gastrointestinal Motility and Metabolic Pharmacoepidemiology Group (2024). Shifting trends in the indication of glucagon-like peptide-1 receptor agonist prescriptions: a nationwide analysis. Ann Intern Med.

[3] Persson C., Eaton A., Mayrovitz H.N. (2025). A closer look at the dermatological profile of GLP-1 agonists. Diseases.

[4] El-Amawy H.S. (2026). Tirzepatide in dermatology: cutaneous adverse events, emerging therapeutic roles, and cosmetic implications – a comprehensive review. An Bras Dermatol.

[5] Rzepka P.V., Kaffenberger J.A. (2020). A case of morbilliform drug eruption to dulaglutide. J Clin Aesthet Dermatol.

[6] Kyriakos G., Diamantis E., Memi E., Elefsiniotis I. (2022). An uncommon case of dulaglutide-related morbilliform drug eruption. Cureus.

[7] Ukoha U.T., Pandya A.G., Dominguez A.R., Hall J.C., Hall B.J. (2015). Cutaneous drug eruptions: diagnosis, histopathology and therapy.

[8] Chu M.-T., Chang W.-C., Pao S.-C., Hung S.-I. (2023). Delayed drug hypersensitivity reactions: molecular recognition, genetic susceptibility, and immune mediators. Biomedicines.

[9] Wei B.M., Fox L.P., Kaffenberger B.H. (2024). Drug-induced hypersensitivity syndrome/drug reaction with eosinophilia and systemic symptoms. Part II diagnosis and management. J Am Acad Dermatol.

